# A simple form could prevent authorship issues in Cochrane manuscripts: A cohort study

**DOI:** 10.1002/cesm.12053

**Published:** 2024-04-05

**Authors:** Siv Fonnes, Kristoffer Andresen, Stina Öberg, Jason Joe Baker, Jacob Rosenberg

**Affiliations:** ^1^ Cochrane Colorectal Group Copenhagen University Hospital — Herlev and Gentofte Herlev Denmark; ^2^ Center for Perioperative Optimization, Department of Surgery Copenhagen University Hospital — Herlev and Gentofte Herlev Denmark

**Keywords:** authorship, cohort, editorial policies, publishing, review literature as topic

## Abstract

**Aim:**

We aimed to investigate authorship issues after the implementation of an authorship declaration form in a Cochrane Review Group.

**Methods:**

The Cochrane Colorectal Group uses an authorship declaration form that consists of three parts: (1) manuscript information, (2) documentation for roles according to the four authorship criteria of the International Committee of Medical Journal Editors (ICMJE), and (3) identification information of individual authors and signed approval. The manuscripts' contact authors were responsible for collecting the forms from all coauthors. This observational cohort study reports on all authorship issues in authorship declaration forms collected from February 2020 to December 2023.

**Results:**

We received 276/277 authorship declaration forms or replies from authors (response rate 99.6%) from 44 manuscripts, including 52% protocols and 48% reviews. There were authorship issues present in 14/44 (32%) of the manuscripts, and the most common issue was that not all authors fulfilled all four ICMJE authorship criteria. Six gift authors were removed from by‐lines. Issues in nine of the 14 manuscripts were resolved by the author group when informing them about the ICMJE authorship criteria and guidance from the Committee on Publication Ethics (COPE). The issues in the remaining five manuscripts were unresolved since the manuscripts were transferred or rejected, thus, ceased to be developed by the Cochrane Colorectal Group.

**Conclusion:**

Authorship issues were raised in almost one‐third of manuscripts. Most issues were resolved and six gift authorships were prevented. The awareness of authorship criteria is sharpened when all authors are individually asked to fill out and sign a form. This could help decrease the rate of unethical authorships in Cochrane publications and contribute to more ethical and robust evidence production.

## 1 INTRODUCTION

**Figure 1:**
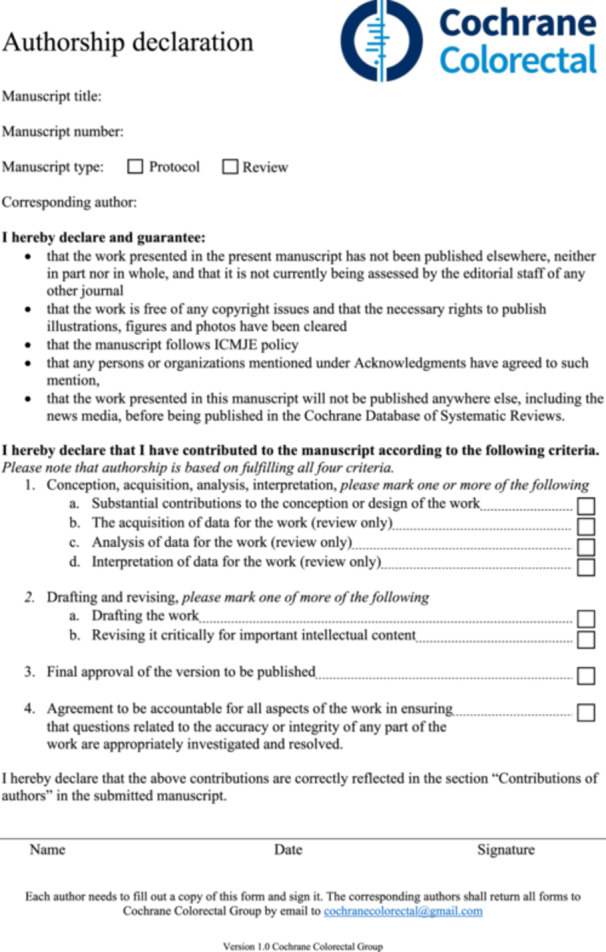
The authorship declaration form, which is also available online [8].

Cochrane reviews are publications of high quality due to their strong methodology [1, 2, 3, 4, 5, 6]. Publishing with Cochrane can lead to both scientific rewards through continuous citations over many years [7] and personal rewards. Therefore, Cochrane publications are susceptible to unethical authorships such as ghost and gift authorships. A gift author refers to a person who is listed as an author but has not contributed to all four mandatory authorship criteria (Figure 1) established by the International Committee of Medical Journal Editors (ICMJE) in 2013 [9]. The fourth criterion regarding accountability was added to ensure that all authors must help in resolving questions of scientific misconduct [10]. Cochrane adheres to these four criteria [11] that play an important part in maintaining research integrity and transparency [12, 13]. However, a recent survey of first authors found that unethical authorships still exist in Cochrane reviews, as 41% of Cochrane reviews had gift authors on the by‐line [14]. To overcome this, Cochrane Colorectal Group initiated the use of an authorship declaration form. This form had to be completed and signed by each author of a Cochrane manuscript before continuing to peer review.

In this study, we aimed to investigate authorship issues after the implementation of an authorship declaration form in a Cochrane Review Group.

## 2 METHODS

This observational cohort study was based on prospectively collected data from Cochrane manuscripts that were under development in the Cochrane Colorectal Group. The study was reported according to STrengthening the Reporting of OBservational studies in Epidemiology (STROBE) statement [15]. Approval from the local Ethics Committee was not required according to Danish legislation [16]. Data are shared but presented in an anonymized form, so authors cannot be identified [17].

### 2.1 Setting and participants

The Cochrane Colorectal Group helps authors develop their Cochrane protocols and reviews before they are submitted for peer review. This process typically consists of (1) an initial check, (2) editorial evaluation, and (3) revision(s). In 2020, an authorship declaration form was initiated during the initial check. Data were collected from February 2020 until the end of December 2023 (convenience sampling).

We included all manuscripts, both protocols and reviews, under development in the Cochrane Colorectal Group. The authorship declaration form [8] (Figure 1), was sent to the contact author. The form consists of three parts: (1) manuscript information, (2) specific documentation for roles according to the four ICMJE authorship criteria [9], and (3) identification information and signed and dated approval by the individual author. The contact author was responsible for returning the authorship declaration form from each author on the by‐line. We included all received authorship declaration forms and correspondences concerning this, regardless of whether the author remained on the by‐line or not and whether the manuscript continued to peer review or not. Finally, all the marked roles were cross‐checked with the “Contribution of authors” section in the manuscripts by the editorial base to ensure that accurate documentation of the roles was available in the manuscript.

### 2.2 Variables and data sources

One author extracted data. The type of manuscript (protocol/review) was extracted from Archie [18]. The number of authors on the by‐line for each manuscript was extracted either from Archie [18] or RevMan Web [19]. The outcome was authorship issues (yes/no), and this was extracted from authorship declaration forms and/or correspondences between the Cochrane Colorectal Group and the contact author, including a brief description. Finally, we assessed whether the authorship issues were resolved or not.

### 2.3 Statistical methods

Most data were dichotomous and reported as numbers and proportions in percent, including a 95% confidence interval for subgroups created in SAS Studio (3.8 Enterprise Edition). For continuous data, for example, the number of authors, we inspected histograms and Q–Q plots. As data were not normally distributed, the median and range were reported.

## 3 RESULTS

All but one author replied by filling out the authorship declaration form or through correspondence with the Cochrane Colorectal Group (response rate 99.6%). Thus, we included 276 authorship declaration forms or replies. There was a median of six authors per manuscript (range = 2−13) on the by‐line of the 44 Cochrane manuscripts in development, consisting of 52% protocols and 48% reviews.

### 3.1 Authorship issues

**Figure 2:**
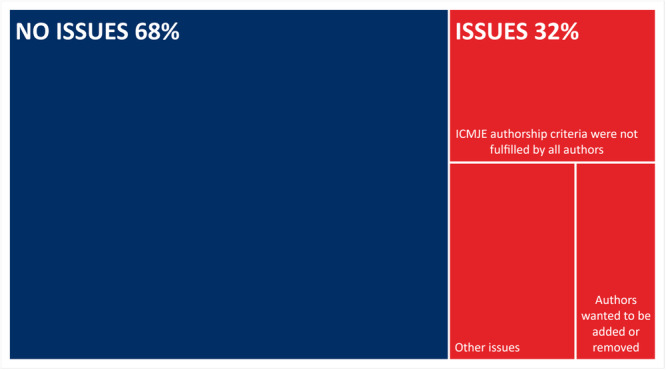
A tree map of the assessment of the received authorship declaration forms and replies for Cochrane protocols and reviews (*n* = 44) and whether authorship issues were present or not. ICMJE: International Committee of Medical Journal Editors [9]. Other issues: Issues present (ICMJE criteria were not fulfilled by all authors and/or missing forms) but unresolved as the manuscript ceased to be handled by the Cochrane Colorectal Group.

An overview of authorship issues for the 44 Cochrane protocols and reviews is presented in Figure 2. There were no authorship issues in 30/44 manuscripts (68%), and issues present in 14/44 manuscripts (32%).

Authorship issues in nine of the 14 manuscripts were resolved. For six of these manuscripts, not all authors fulfilled all four ICMJE authorship criteria when the initial form was received. In two manuscripts, four and two authors, respectively, wished to be removed from the by‐line. In one manuscript, authors wanted to be added to the by‐line. Cochrane Colorectal Group raised all issues to the authors and informed them of both the ICMJE criteria for authorship [9] and the Committee On Publication Ethics (COPE) guidance [20], and the author group resolved the issues accordingly. Five of the 14 manuscripts with issues present remained unresolved as the manuscript ceased to be handled by the Cochrane Colorectal Group due to either transfer to another Cochrane Review Group or rejection for various reasons [15].

The proportions of authorship issues were similar in most subgroups, Table 1.

**Table 1:** Authorship issues for different subgroups are presented as number (*n*) and proportions with 95% confidence intervals (CI).

Subgroup	*n* authorship issues/*n* total	% (95% CI)	*p* value
Number of authors			
<5 authors	2/10	20% (3%–56%)	See legend^a^
5−8 authors	6/25	24% (9%–45%)	
>8 authors	6/9	67% (30%–93%)	
Type of manuscript			
Cochrane protocols	8/23	35% (16%–57%)	0.75
Cochrane reviews	6/21	29% (11%–52%)	
Region			
Asia or Oceania	5/16	31% (11%–59%)	
Europe	9/28	32% (16%–52%)	1.0

*Note*: *p* value from *χ* ^2^ test was used unless stated otherwise. Geographic regions were based on the contact author and United Nations definition (https://unstats.un.org/unsd/methodology/m49/). The results were: <5 authors versus 5–8 authors *p* = 1.0, <5 authors versus >8 authors *p* = 0.07, and 5−8 authors versus >8 authors *p* = 0.04.

^a^ Tested with Fisher's exact test as number of expected counts were <5.

## 4 DISCUSSION

After the implementation of an authorship declaration form, issues were raised in one‐third of Cochrane manuscripts under development. The most common issue was that authors did not fulfill all four ICMJE authorship criteria. Furthermore, six gift authorships and one ghost authorship were possibly prevented because of the authorship form.

We detected authorship issues in one‐third of the Cochrane protocols and reviews. This is lower than in a survey of first authors of Cochrane reviews, where 41% of the reviews had gift authors [14]. Authorship issues are not only present in Cochrane publications. A meta‐analysis estimated that 51% of the surveyed publications had gift authors when comparing contributions and ICMJE criteria [21], and a survey of corresponding authors in different journals found 40% gift authors [22]. However, a lower prevalence (9%−13%) has been reported in other journals [23, 24]. Unfortunately, most surveys suffer from a low response rate [21, 24].

The most common authorship issue in this study was that not all authors fulfilled all four authorship criteria, and an increased focus on these seems warranted. The criteria were inadequately documented in both Cochrane reviews with group authorships [25] and with more than 15 authors [26]. Furthermore, it seems that not all ICMJE authorship criteria are regarded equally important by authors. Most corresponding authors regard the first criterion (concept/design, acquisition, analysis, interpretation) and/or the second criterion (drafting or revising) as important [27]. Another survey found that the corresponding authors had never heard of or were unfamiliar with ICMJE authorship criteria in 25% of manuscripts submitted in 2016 to the BMJ Publishing Group [28].

The strength of this study includes its prospective design and the use of a standardized authorship declaration form that followed the format of the internationally recognized ICMJE criteria for authorship [9]. Furthermore, each author personally had to mark whether they had performed the specific tasks and sign the form instead of simply confirming that they fulfilled the criteria, for example, CRediT [29], as provided by some editorial management systems. The editorial base acted after receiving the forms. They thoroughly checked them, followed up on issues, and ensured that the author contribution section of the manuscript and the final authorship declaration form were aligned. The high response rate implied that the authors acknowledged the form and that any authorship issues had to be resolved before further development of the manuscript. Limitations include that a small number of submissions were evaluated and that the performed roles were self‐reported by the authors. Thus, roles may be overreported due to social desirability bias. Also, the rate of authorship issues may have been underestimated since data were not collected anonymously. This could have affected the responses because the authorship declaration forms were evaluated by the Cochrane Colorectal Group, who can reject Cochrane protocols and reviews in development. Furthermore, we did not ask authors if they found that it prevented authorship issues, and the authorship declaration form cannot detect ghost authors.

Structural initiatives exist [30, 31], but their effect is difficult to evaluate. We recommend that this authorship declaration form is implemented. It is easy to administer, and it resulted in authors being removed from the by‐line, thus lowering the rate of gift authors. It was relevant to implement during the development process of manuscripts for both protocols and reviews before peer review was conducted. We plan to follow up with a survey of authors to investigate if the authorship declaration form increases their awareness of the ICMJE authorship criteria and authorship issues.

In conclusion, authorship issues were raised in almost one‐third of submissions, and all were subsequently resolved for Cochrane Colorectal Group's manuscripts. The awareness of authorship criteria seems to be sharpened when using an authorship declaration form, and some gift authorships were prevented. Utilizing an authorship form can help decrease the rate of unethical authorships in Cochrane publications and contribute to increased ethical and robust evidence production.

## AUTHOR CONTRIBUTIONS

**Siv Fonnes**: Conceptualization; data curation; formal analysis; writing—original draft. **Kristoffer Andresen**: Conceptualization; data curation; writing—review and editing. **Stina Öberg**: Data curation; writing—review and editing. **Jason Joe Baker**: Data curation; writing—review and editing. **Jacob Rosenberg**: Conceptualization; writing—review and editing.

## CONFLICT OF INTEREST STATEMENT

All authors report a relationship with Cochrane Colorectal Group that includes: employment. No other potential conflict of interest.

## PEER REVIEW

The peer review history for this article is available at https://www.webofscience.com/api/gateway/wos/peer-review/10.1002/cesm.12053.

## Data Availability

Data are available from https://doi.org/10.5281/zenodo.10530504 but in anonymized form.

## References

[1] Fleming PS , Seehra J , Polychronopoulou A , Fedorowicz Z , Pandis N . Cochrane and non‐Cochrane systematic reviews in leading orthodontic journals: a quality paradigm? Eur J Orthod. 2013;35:244‐248. 22510325 10.1093/ejo/cjs016

[2] Windsor B , Popovich I , Jordan V , Showell M , Shea B , Farquhar C . Methodological quality of systematic reviews in subfertility: a comparison of Cochrane and non‐Cochrane systematic reviews in assisted reproductive technologies. Hum Reprod. 2012;27:3460‐3466. 23034152 10.1093/humrep/des342

[3] Wen J , Ren Y , Wang L , et al. The reporting quality of meta‐analyses improves: a random sampling study. J Clin Epidemiol. 2008;61:770‐775. 18411041 10.1016/j.jclinepi.2007.10.008

[4] Moher D , Tetzlaff J , Tricco AC , Sampson M , Altman DG . Epidemiology and reporting characteristics of systematic reviews. PLoS Med. 2007;4:e78. 17388659 10.1371/journal.pmed.0040078 PMC1831728

[5] Delaney A , Bagshaw SM , Ferland A , Laupland K , Manns B , Doig C . The quality of reports of critical care meta‐analyses in the Cochrane Database of Systematic Reviews: an independent appraisal. Crit Care Med. 2007;35:589‐594. 17205029 10.1097/01.CCM.0000253394.15628.FD

[6] Jørgensen AW , Hilden J , Gøtzsche PC . Cochrane reviews compared with industry supported meta‐analyses and other meta‐analyses of the same drugs: systematic review. BMJ. 2006;333:782. 17028106 10.1136/bmj.38973.444699.0B PMC1602036

[7] Hoffmeyer B , Fonnes S , Andresen K , Rosenberg J . Use of inactive Cochrane reviews in academia: a citation analysis. Scientometrics. 2023;128:2923‐2934.

[8] Cochrane Colorectal Group . *Authorship Declaration Form*. Accessed March 14, 2024. https://colorectal.cochrane.org/new-authors/authorship-declaration

[9] International Committee of Medical Journal Editors . *Defining the Role of Authors and Contributors*. Accessed March 14, 2024. http://www.icmje.org/recommendations/browse/roles-and-responsibilities/defining-the-role-of-authors-and-contributors.html

[10] Rosenberg J , Bauchner H , Backus J , et al. The new ICMJE recommendations. N Z Med J. 2013;126:9‐11. 24150260

[11] Cochrane . *Editorial policies*. Accessed March 14, 2024. https://www.cochranelibrary.com/cdsr/editorial-policies

[12] World Conferences on Research Integrity . *Singapore Statement on Research Integrity*. Accessed March 14, 2024. https://www.wcrif.org/guidance/singapore-statement

[13] Cochrane . Research Integrity. Accessed March 14, 2024. https://community.cochrane.org/organizational-info/resources/research-integrity

[14] Gülen S , Fonnes S , Andresen K , Rosenberg J . More than one‐third of Cochrane reviews had gift authors, whereas ghost authorship was rare. J Clin Epidemiol 2020;128:13‐19. 32781115 10.1016/j.jclinepi.2020.08.004

[15] von Elm E , Altman DG , Egger M , et al. The strengthening the reporting of observational studies in epidemiology (STROBE) statement: guidelines for reporting observational studies. Lancet 2007;370:1453‐1457. 18064739 10.1016/S0140-6736(07)61602-X

[16] Capital Region of Denmark . *What Must I Report?* Accessed March 14, 2024. https://www.regionh.dk/til-fagfolk/forskning-og-innovation/de-regionale-videnskabsetiske-komiteer/sider/hvilke-projekter-skal-jeg-anmelde.aspx

[17] Fonnes S , Andresen K , Öberg S , Baker JJ , Rosenberg J . *Data on Authorship Issues in Cochrane Manuscripts*. Accessed March 14, 2024. 10.5281/zenodo.10530504 PMC11795933 40474905

[18] Cochrane . *Archie*. Accessed March 14, 2024. https://training.cochrane.org/online-learning/core-software-cochrane-reviews/archie

[19] Cochrane . *RevMan Web*. Accessed March 14, 2024. https://revman.cochrane.org/info

[20] Committee on Publication Ethics (COPE) . *Flowcharts*. Accessed March 14, 2024. https://publicationethics.org/guidance/Flowcharts?t=authorship&sort=score

[21] Meursinge Reynders RA , Ter Riet G , Di Girolamo N , Cavagnetto D , Malički M . Honorary authorship is highly prevalent in health sciences: systematic review and meta‐analysis of surveys. Sci Rep. 2024;14:4385. 38388672 10.1038/s41598-024-54909-w PMC10883936

[22] Flanagin A . Prevalence of articles with honorary authors and ghost authors in peer‐reviewed medical journals. JAMA. 1998;280:222‐224. 9676661 10.1001/jama.280.3.222

[23] Vinther S , Rosenberg J . Appearance of ghost and gift authors in Ugeskrift for Læger and Danish Medical Journal. Danish Med J. 2012;59:4455. 22549492

[24] Rietdijk W , Mandigers L , Bakker J . Do perceived honorary authors influence publication chance? Survey evidence from the Journal of Critical Care. J Crit Care. 2020;60:202‐208. 32871417 10.1016/j.jcrc.2020.05.010

[25] Andersen MZ , Fonnes S , Andresen K , Rosenberg J . Group authorships in Cochrane had low compliance with Cochrane recommendations. Journal of Evidence‐Based Medicine. 2020;13:199‐205. 32558203 10.1111/jebm.12396

[26] Gülen S , Fonnes S , Andresen K , Rosenberg J . Increasing number of authors in Cochrane reviews. J Evidence‐Based Med. 2020;13:34‐41. 10.1111/jebm.12371 32086993

[27] Malički M , Jerončić A , Marušić M , Marušić A . Why do you think you should be the author on this manuscript? Analysis of open‐ended responses of authors in a general medical journal. BMC Med Res Methodol. 2012;12:189. 23256648 10.1186/1471-2288-12-189 PMC3552823

[28] Schroter S , Montagni I , Loder E , Eikermann M , Schäffner E , Kurth T . Awareness, usage and perceptions of authorship guidelines: an international survey of biomedical authors. BMJ Open. 2020;10:e036899. 10.1136/bmjopen-2020-036899 PMC7507845 32958486

[29] Allen L , Scott J , Brand A , Hlava M , Altman M . Publishing: credit where credit is due. Nature. 2014;508:312‐313. 24745070 10.1038/508312a

[30] All European Academies . *The European Code of Conduct For Research Integrity*. Accessed March 14, 2024. https://ec.europa.eu/info/funding-tenders/opportunities/docs/2021-2027/horizon/guidance/european-code-of-conduct-for-research-integrity_horizon_en.pdf

[31] United Kingdom Research Integrity Office . *Code of Practice for Research*. Accessed March 14, 2024. https://ukrio.org/wp-content/uploads/UKRIO-Code-of-Practice-for-Research.pdf

